# Neuroimaging advances in neurocognitive disorders among HIV-infected individuals

**DOI:** 10.3389/fneur.2025.1479183

**Published:** 2025-02-13

**Authors:** Han Wang, Xiaolin Jiu, Zihua Wang, Yanwei Zhang

**Affiliations:** ^1^Department of Radiology, Bethune International Peace Hospital (the 980th Hospital of PLA Joint Logistic Support Force), Shijiazhuang, Hebei, China; ^2^Department of Radiology, Hebei Medical University, Shijiazhuang, Hebei, China; ^3^Department of Oncology, Bethune International Peace Hospital (the 980th Hospital of PLA Joint Logistic Support Force), Shijiazhuang, Hebei, China

**Keywords:** HIV-associated neurocognitive disorders, functional magnetic resonance imaging, structural magnetic resonance imaging, diffusion magnetic resonance imaging, quantitative magnetic resonance imaging

## Abstract

Although combination antiretroviral therapy (cART) has been widely applied and effectively extends the lifespan of patients infected with human immunodeficiency virus (HIV), these patients remain at a substantially increased risk of developing neurocognitive impairment, commonly referred to as HIV-associated neurocognitive disorders (HAND). Magnetic resonance imaging (MRI) has emerged as an indispensable tool for characterizing the brain function and structure. In this review, we focus on the applications of various MRI-based neuroimaging techniques in individuals infected with HIV. Functional MRI, structural MRI, diffusion MRI, and quantitative MRI have all contributed to advancing our comprehension of the neurological alterations caused by HIV. It is hoped that more reliable evidence can be achieved to fully determine the driving factors of cognitive impairment in HIV through the combination of multi-modal MRI and the utilization of more advanced neuroimaging analysis methods.

## Introduction

Due to the combination antiretroviral therapy (cART), acquired immunodeficiency syndrome (AIDS), caused by human immunodeficiency virus (HIV), has been transformed from a rapidly fatal disease disease to a manageable chronic condition (1, 2). The acquired cognitive impairment, referred to as HIV-associated neurocognitive disorders (HAND), is one of the major concerns for the aging population of people living with HIV (3). Currently, three classification categories of HAND have been proposed: asymptomatic neurocognitive impairment (ANI), HIV-associated mild neurocognitive disorder (MND), and HIV-associated dementia (HAD) (4). The incidence of ANI and MND remains prevalent and may be on the rise despite successful viral suppression (5–8). Furthermore, research has shown that nearly half of the patients experience neurocognitive impairment (7).

HIV is a retrovirus that attacks the human immune system, causing a decrease in the CD4^+^ T cells and leading to immune deficiency (9, 10). The exact mechanisms underlying HAND remain incompletely understood, with various hypotheses putting forth. The blood-brain barrier (BBB) is crossed by HIV within days of infection, with the virus infiltrating the central nervous system (CNS) (11) and causing neuronal and glial damage through the release of viral proteins, cytokines, and immune cell secretions (12–14). Recent research found intact provirus in the frontal white matter, indicating a complete and potentially replicating HIV reservoir within the CNS (15, 16). Neurotoxic effects of cART also have been noted (17, 18), leading to the development of HAND by reducing neuronal axon length (19), mitochondrial DNA content (20), and dendritic spines on neurons (21). Both HIV and cART can influence oxidative stress, gene expression, and various cellular signal transduction pathways. Additionally, neurotoxins induced by HIV and elevated metabolic products can disrupt the homeostasis of the neural microenvironment (22). Overall, cognitive decline in patients with HIV may result from the synergistic effects of multiple factors.

With the advent and advancement of neuroimaging, the non-invasive nature and capacity to visualize brain functional/structural changes have facilitated the investigation of neural mechanisms of HAND. Magnetic resonance imaging (MRI) techniques, including functional MRI, structural MRI, diffusion MRI, and quantitative MRI, have increased utility in detecting and managing HAND. This review aims to summarize recent studies performed neuroimaging analysis in individuals with HAND.

## Functional magnetic resonance imaging (fMRI)

Blood oxygen level-dependent fMRI (BOLD-fMRI) is one of the most used techniques in studies on HAND. It reveals the coupling between neural activity and cerebral blood flow (23, 24). BOLD-fMRI consists of two types: resting-state fMRI (rs-fMRI) and task-related fMRI.

### Resting-state fMRI

The rs-fMRI measures spontaneous low-frequency fluctuations in the BOLD signal to investigate the functional alternations of the brain in the absence of a task or stimulus (25). Frequently used analyzing methods in HAND studies can be grouped into three categories: local voxel-wise indices, functional connectivity (FC), and graph theory.

The local voxel-wise indices mainly include the amplitude of low-frequency fluctuation (ALFF)/fractional amplitude of low-frequency fluctuation (fALFF) (26) and regional homogeneity (ReHo) (27). All these indicators depict the features of the BOLD signal in a single voxel or neighboring voxels. Most studies indicate lower ALFF/fALFF and ReHo in the frontal lobe, temporal lobe, occipital lobe, and hippocampus (28–32) in people living with HIV (PLWH), which is associated with cognitive decline. However, recent research has reported higher fALFF in the occipital lobe with patients who exhibit cognitive impairment (33), as well as higher ReHo and ALFF in the medial orbital lobe of perinatally HIV-infected patients (PHIV) (34). Additionally, these indexes were used to evaluate the efficacy of cART. HIV+/cART+ individuals were found to have increased ALFF in the right superior temporal gyrus and supramarginal gyrus compared to patients without treatment (35). The occipital lobe and orbitofrontal cortex have been reported to be the most susceptible regions to HIV-related effects, with a decrease in spontaneous activity observed. The increase of rs-fMRI indices may reflect a compensatory mechanism of the brain. Similarly, improvements in brain function and cognitive performance have been noted following the implementation of cART.

Unlike the local voxel-wise indexes, the FC measures the synchronization between brain regions/voxels (36). This connectivity is hypothesized to be disrupted by HIV, which is associated with a decline in cognitive function (37, 38). Increased FC has also been detected among several brain regions in patients with cognitive impairments, including the right lingual gyrus and right middle occipital gyrus (29), the right cerebellar lobule VI and the anterior cingulate cortex (39), as well as the right superior occipital gyrus and right olfactory cortex (33). Additionally, studies have indicated that the improvement in cognitive function is attributed to the recovery of FC by cART (40, 41). However, a study by Thippabhotla et al. (42) observed no significant differences in FC between HIV+ and HIV- individuals, nor between cognitively impaired and cognitively normal patients with HIV. They indicate factors such as sample size (43) or global signal regression (GSR) may lead to paradoxical perspectives. GSR remains a controversial issue (44). Therefore, it is essential to verify whether GSR has been utilized in the analysis when interpreting comparative results to avoid potential misinterpretations.

Aging and sleep disorders may act as the mediating factors that affect FC between brain regions, thereby exacerbating cognitive impairments (45, 46). In contrast to HIV seronegative individuals, FC tends to decrease with age in patients infected with HIV (47, 48), particularly within the default mode network (DMN) and the salience network (SN) (49). However, Brandon et al. (50) observed that FC generally increases with age and further increases with HIV infection, indicating that aging and HIV are identified as independent influencing factors. Sleep disturbances, which are common accompaniments of aging and a risk factor for Alzheimer's disease (51), have been found to exhibit a 100% prevalence in individuals with HAND (52). These disturbances are linked to impaired clearance by the glymphatic system (53). Correlations were identified by Venkataraman et al. (46) between the activity levels of the DMN, the frontoparietal network, and the SN with scores on the Pittsburgh Sleep Quality Index (PSQI). A progressive interrelationship among HIV, aging, and sleep disturbances enhances the risk of cognitive impairment in PLWH.

A common assumption in most rs-fMRI studies is temporal stationarity, which overlooks the inherently dynamic and non-linear features of the brain across different times (54). To overcome this limitation, researchers have introduced advanced methods such as dynamic functional connectivity (dFC) (55, 56) and mutual connectivity analysis (MCA) (57). The study observed enhanced dFC variability between the left superior frontal and right inferior frontal gyrus in patients with ANI (58). AM and colleagues reported MCA can discriminate between PLWH and healthy controls, with increased interaction strength observed in the basal ganglia and frontal cortex of patients with HAND (59, 60). Whereas, Abidin et al. (61) noted disconnections within frontal and occipital cortices compared to the healthy group. Despite these findings, research on dynamic analysis remains limited. There is a need for further research to identify the changes in dynamic brain function associated with varying disease durations and viral loads.

While the FC method only takes into account a limited number of brain regions, researchers hope to obtain more comprehensive information regarding the whole brain functional network. The graph theory elucidates the topological structure and efficiency of the brain network (62). In functional networks, nodes correspond to different brain regions, and edges denote the FC between these regions. Studies have demonstrated that the topology of the brain functional network experiences a subtle reorganization, leading to a significant decrease in the modularity and small-world characteristics (63, 64) associated with executive function (65). Regarding HIV and aging, it has been established that HIV correlates with lower closeness centrality and eigenvector centrality, while age is related to higher graph entropy (66).

### Task-related fMRI

Task-related fMRI means the fMRI data is collected while individuals perform various tasks designed to activate and identify functional brain parcels (67). Few studies have reported deficits in attention and memory functions, accompanied by enhanced utilization of brain reserve areas in PLWH (68–71). A longitudinal study revealed increased activation in the prefrontal and posterior parietal cortices compared to 1 year earlier after stable cART. Conversely, a decrease in activation was observed in the corresponding regions in HIV-seronegative individuals (72). These indicate a decline in neural processing efficiency and suggest ongoing brain damage. Task-related fMRI was more commonly used earlier, but recent studies have decreased. Future studies should integrate task-related fMRI with rs-fMRI to investigate changes in brain function.

Overall, fMRI is a powerful technique that offers a wealth of information about brain activity. It is anticipated that future research will benefit from the integration of novel technologies and methodologies, which will facilitate a comprehensive investigation of HIV.

## Structural magnetic resonance imaging (sMRI)

The brain sMRI assesses the neuroanatomic changes associated with HIV infection using T1-weighted MRI (73, 74). The analyzing approaches mainly comprise voxel-based morphometry, surface-based morphometry, and structural covariance network.

### Volume-based morphometry

The volume-based morphometry method performs unbiased measurement and analysis of every voxel in the volumetric data other than the segmented region of interest (74). Voxel-based morphometry (VBM), deformation-based morphometry (DBM), and tensor-based morphometry (TBM) are the detailed strategies utilized in HAND studies.

The VBM is primarily used for assessing the volumes of gray and white matter (75). Significant atrophy in the volumes of the thalamus, hippocampus, frontal lobes, and occipital lobes, along with a notable increase in the volume of the third ventricle, brainstem, putamen, and lateral ventricles, has been observed in patients with intact cognition (38, 76–80). Subsequent research identified atrophy in the anterior cingulate cortex, temporal lobes (81), striatum (82), and cerebellum (83) of patients with HAND, with the severity of atrophy intensifying as cognitive impairments worsen. Additionally, brain volume loss has been correlated with CD4 cell counts (81, 84–86) and viral load (87). In conclusion, brain volume alterations are characterized as a feature associated with HIV infection.

The PHIV patients constitute a unique population considering the relatively long course of the disease and the tendency to receive early treatment. Studies have demonstrated they experience cognitive deficits and developmental delays that may extend into adulthood with a reduction in brain capacity, even with the early initiation of cART and effective control of viral load (88–91). However, Sarma et al. (92) observed no significant differences in the global volumes compared to the control group, while there were regional alterations in gray and white matter. Further, longitudinal studies are needed to describe the active ongoing brain infection or toxicity from HIV treatment.

The TBM and DBM are based on the deformation fields obtained by non-linear registration of brain images. TBM focuses on describing variations in the local shape of brain structures (93), while DBM is concerned with detecting differences in the relative positioning of brain structures (94). Atrophy in the striatum and frontoparietal lobes of PLWH has been observed and may serve as a precursor to HAD (95). Longitudinal studies have been designed to investigate whether cART can halt the progression of brain injury associated with HIV. Sanford et al. indicate that changes in brain volume and cognitive ability over 2 years were comparable between individuals with HIV after cART and those without HIV (96, 97). Another study has reported significant atrophy in the putamen, caudate nucleus, and hippocampus over a 2-year period, suggesting this atrophy may exceed the effects associated with aging despite the immediate initiation of cART during acute infection (98). Comprehensive analysis in combination with other methods is needed in the future.

### Surface-based morphometry

The SBM examines the geometric properties of the cerebral cortex surface to identify structural changes and can provide insights into cortical thickness and cortical surface area (99). Most studies have revealed that reduced cortical thickness in the cingulate, frontal, and temporal lobes in PLWH is associated with cognitive decline (84, 85, 100). Similarly, decreases in cortical thickness, cortical surface area, and gyrification are associated with peak viral loads and nadir CD4% among PHIV youth (101–103). However, other studies have revealed that the developmental trajectories of cortical thickness and gyrification in PHIV patients who initiate treatment early are similar to those without HIV (104). Additionally, intentional interruptions of cART have not been found to have significant impacts on cortical morphological development (105, 106). A slight increase in the cortical thickness of the frontal and temporal lobes has also been observed with the prolongation of treatment (97). These findings suggest that early initiation of cART exerts a restorative influence on the structural damage of the brain in PLWH. Studies integrating volume-based analysis with surface-based analysis have also observed reductions in subcortical volumes and cortical thickness among PLWH (84, 96). Larger cohort studies with extended follow-up periods are warranted to further investigate the impact of HIV and the role of cART on brain structural alternations.

Fractal dimension (FD) calculated on the cortical surface provides a quantitative description of the structural complexity in the cerebral cortex, which is challenging to assess by standard morphometric techniques (107, 108). It has been observed that the cerebral cortex initiates a thinning pattern when the FD is increased. FD also has a significant relationship with intelligence and education levels (107). A study by Weber et al. (109) has demonstrated that higher FD in the caudate nucleus, hippocampus, frontal lobe, and occipital lobe correlates with better cognitive performance. FD may be more sensitive in identifying brain damage compared to cortical thickness or volumes of gray matter/white matter.

### Structural covariance network

The structural network can be described as graphs that are composed of nodes indicating neural elements (neurons or brain regions) that are linked by edges representing physical connections (synapses or axonal projections) (62, 110). A subtype of the structural network, the structural covariance network (SCN), is characterized by the covariation in structural features (including gray matter volume, cortical thickness, and cortical surface area) across different brain regions (111). The SCN is constructed based on gray matter volume that demonstrates greater centrality within the prefrontal cortex, as well as diminished centrality and nodal path length within the temporal and frontal lobes of PLWH. These findings suggest a potential disruption in the anatomical connections among brain regions (112, 113). Subsequently, further research found a reduction in betweenness centrality of the anterior cingulate cortex of PLWH with ANI (114), which may intensify as the disease progresses.

In short, sMRI enables the assessment of brain structural changes through a range of indicators, serving as a potential marker for the early detection of cognitive decline in PLWH.

## Diffusion magnetic resonance imaging (dMRI)

Diffusion magnetic resonance imaging (dMRI) is primarily employed to evaluate the lesion of microstructure in the white matter (115–117). Typical techniques include diffusion tensor imaging (DTI), diffusion kurtosis imaging (DKI), diffusion spectrum imaging (DSI), and high-angular resolution diffusion imaging (HARDI), with increasing capacity to map complex fiber architectures in tissues. Based on the disparities in metrics obtained from the diffusion data, we categorize the relevant studies into three types.

### Tractography-based spatial statistics

Voxel-based analysis (VBA) serves as the common analytical method, involving the registration of all images into a standardized space, subsequently subjected to voxel-wise statistical analysis. Tract-based spatial statistics (TBSS), as a complementary method to VBA, performs statistical analyses on a generated white matter skeleton and overcomes the limitations, such as alignment inaccuracies and the lack of a principled approach to selecting the extent of smoothing (118). Fractional anisotropy (FA), mean diffusivity (MD), axial diffusivity (AD), and radial diffusivity (RD) can be calculated; changes in these metrics indicate inflammation and microstructural abnormalities (119–122).

HIV infection induces dynamic alterations in the brain's microstructure, and these changes tend to exacerbate as the disease progresses. Increased FA was observed in the corpus callosum of individuals with primary HIV infection, as well as lower FA and higher MD were found across extensive brain regions in patients who have been ill for longer (123–129). Furthermore, an increase in AD is noted in patients with HAND (130, 131). This increase in FA may indicate a compensatory mechanism, implying that early damage to axonal and myelin in the brain's white matter could occur during the initial stages of HIV infection, albeit to a minor degree. With the prolongation of the disease duration, the compensatory mechanisms are insufficient to offset the effects of HIV, leading to significant damage to white matter.

The neuroprotective effects of cART have been assessed by examining the integrity of the white matter microstructure. Sarma et al. (132) reported increased FA in the left middle frontal gyrus and right precuneus of PHIV youth. However, some researchers consider that although cART can effectively suppress viral replication, it cannot completely restore the compromised white matter microstructure. A longitudinal study on rhesus monkeys revealed decreased FA and increased MD in periventricular structures (133), with these microstructural alterations persisting and progressing over time, even with cART (134). These differences may be related to the species, the duration of cART, serum viral load, and CD4^+^ T cell levels.

The studies above utilize the DTI based on the Gaussian model. DKI, an advancement based on DTI, quantifies the deviation of water molecule diffusion from a normal distribution within tissues (135–137). Garaci et al. (138) discovered that the nadir-CD4 and the %CD4 exhibited positive correlations with kurtosis anisotropy (KA) and FA, and negative correlations with MD in PLWH. The neurite orientation dispersion and density imaging (NODDI) is a biophysical model that can be fitted using data compatible with clinically feasible scan times (139). By constructing the NODDI model, researchers have recently discovered that PLWH exhibit a lower FA and neurite density index (NDI) (140). This finding indicates a more extensive pattern of brain damage relative to that identified by conventional DTI models.

### Fixel-based analysis

Fixel-Based Analysis (FBA) can describe fiber bundles of varying orientations within a voxel and offers more detailed information by calculating fiber density and fiber cross-sectional area (141, 142). Finkelstein et al. (143) observed a decrease in both fiber density and fiber cross-sectional area within the posterior limb of the internal capsule, the superior corona radiata, and the cerebellar peduncles. However, Zhao et al. (144) identified significant differences only in fiber density, with an increase observed in bilateral frontoparietal bundles, frontal corona radiata, and left arcuate fasciculus in PLWH. More studies that focus on PLWH in FBA are needed in the future.

### Structural connectivity network

Graph-theoretic and dMRI-based tractography approaches were used to investigate the topological organization of PLWH. Structural connectivity (SC) in brain networks refers to the physical connections that are established between different brain regions through white matter fiber tracts (145), employing two primary approaches: deterministic tractography (146) and probabilistic tractography (147). Several studies suggest disruptions in the integrity of the brain network among HIV+ patients. These disruptions are characterized by lower clustering coefficients, which are related to cognitive impairment and nadir CD4 count (148–150). Aili et al. (151) found abnormal connections in HIV+ patients were mainly located in the occipital lobe and parietal lobe, which is consistent with the results of fMRI.

Overall, dMRI is recognized as a sensitive method for evaluating white matter microstructure, with a focus anticipated on future large-sample longitudinal studies utilizing HARDI.

## Quantitative magnetic resonance imaging (qMRI)

The qMRI surpasses conventional MRI by directly yielding specific physical parameters related to the nuclear spin of protons in water (152). Methods such as magnetic resonance spectroscopy (MRS), relaxometry, and arterial spin labeling (ASL) are reported to be employed in studies on HAND.

### Magnetic resonance spectroscopy

MRS enables the measurement of a diverse range of metabolites. These metabolite levels serve as indicators, offering insights into the integrity of neural tissues. Specifically, they can reflect the processes of cell membrane synthesis and renewal, as well as the concentrations of metabolites associated with neuroinflammation (153, 154). The measured metabolites include: (1) n-acetyl aspartate (NAA)- a neuronal marker, (2) choline (Cho)- a membrane marker, (3) creatine (Cr)- a marker for cellular energy metabolism, and (4) myo-inositol (MI)- a marker of gliosis (155, 156).

The concentrations of multiple metabolites have been found to be closely associated with cognitive performance. In patients with HAND, increased Cho and Cr and a concomitant decrease in NAA have been observed in several key brain regions, including the basal ganglia, thalamus, centrum semiovale, and frontal white matter (157–160). Similar findings are documented in chronic conditions (161). These studies indicate that HIV infection can affect a series of processes, including glial cell activation, inflammatory responses, and neuronal metabolism.

MRS is also employed to evaluate the effects of cART on neurons. Elevated ratios of MI/Cr (162) and NAA/Cr (163) have been demonstrated in PLWH receiving cART within the anterior/posterior cingulate cortex, prefrontal cortex, and intraparietal sulcus. A longitudinal research indicates that higher Cr and Cho were observed in children who initiated cART before 12 weeks (164). Other studies have revealed a reduction in NAA among patients with chronically infected despite cART suppressing viral load and reducing brain inflammation (165–169). Damaged neurons can be reshaped by cART. However, it becomes difficult for cART to control the occurrence of neuroinflammation as the disease worsens. Overall, MRS can be more sensitive than conventional MRI in detecting brain abnormalities associated with HIV, and it is likely to offer insight into neurocognitive impairment at the cellular level.

### T1/T2 mapping

Relaxometry entails measuring relaxation times derived from MR images. T1, T2, and T2^*^ values can be estimated using the mapping techniques (170). The T1 relaxation time is sensitive to the organization of molecules within the tissue, while the T2 relaxation time is sensitive to the quantification of paramagnetic substances and provides additional insights into the presence of free and bound water molecules (171, 172). Relaxometry showed significant abnormalities with lengthening of T2 in multiple regions among PLWH, including frontal, parietal, occipital lobes, and basal ganglia (173–175). Recently, Perrotta et al. (172) reported hypointensity of T2, which may be an outcome of reduced demyelination among PLWH receiving Rivastigmine. Therefore, T1 and T2 offer complementary insights into brain tissue structure and its components. Still, research in this field is limited, indicating that future studies could combine with other methods to uncover additional changes.

### Arterial spin labeling

ASL is an MR perfusion technique employing the water in the arterial blood as endogenous tracers (176, 177). The parameter most commonly derived is cerebral blood flow (CBF), which evaluates tissue perfusion quantitatively (178).

Significant variability in normal cerebral perfusion is associated with age and hemodynamic function. A decrease has been indicated predominantly in the lenticular nuclei, posterior cingulate, caudate nucleus, bilateral temporal lobes, occipital lobes, and frontal cortex after HIV infection (179–181), which is associated with factors including age (182–184), viral load (185), physical state (186), and the depletion of CD4^+^ T cells (187). Studies have observed an elevation in CBF within the cortical gray matter, basal ganglia, and thalamus of untreated or perinatally infected patients (188–190). Additionally, longitudinal research detected no significant alterations in CBF among adolescent PHIV (191). These studies imply that such differences might be connected to the dynamic characteristics of CBF. Further longitudinal research is necessary.

## Combination of multimodal neuroimaging methods

The integration of multimodal neuroimaging data is likely to reveal more information, as it can utilize the complementary metrics of different modalities to explore structural and functional characteristics of the brain in both health and disease states.

Studies have demonstrated declined cognitive function in PLWH, and the poor cognitive performance is closely associated with microstructural/macrostructural abnormalities, as well as spontaneous brain activity (38, 77, 79, 192, 193). By integrating multiple methods, Samboju et al. (194) discovered no significant differences in the DTI metrics of individuals with acute HIV infection compared to healthy controls, suggesting that brain integrity is preserved during the acute early stage of HIV infection. Subsequently, Underwood et al. (195) observed extensive microstructural abnormalities in the white matter of PLWH being associated with cognitive impairment in PLWH. These studies may indicate that as AIDS progresses, there is a gradual deterioration of the brain's microstructural integrity.

Recently, researchers have been making efforts to integrate a variety of imaging techniques with MRS. For instance, Khobo et al. (196) combined sMRI, DTI, and MRS to explore brain changes in PHIV patients. Their findings revealed a reduction in the volume of the bilateral globus pallidus, amygdala, and basal ganglia, along with an expansion of the ventricular system at the same time. Moreover, the MD, AD, and RD of white matter fiber tracts, such as the uncinate fasciculus, superior temporal longitudinal fasciculus, and corticospinal tract, exhibited a significant increase. Furthermore, Morgello et al. (197) attempted to predict the characteristics of MRS and diffusion weighted imaging (DWI) by utilizing immune or clinical factors. They discovered that immunological factors related to the duration of the disease could effectively predict MRS metabolites. Meanwhile, the FA and MD values were found to be associated with the disease status of the patients.

## Discussion

Despite the significant global prevalence of PLWH, research into HAND remains constrained, and the underlying mechanisms of the condition are not yet fully elucidated. Neuropsychological assessment tools, which are vital for diagnosing HAND, have inherent limitations that may affect diagnostic accuracy. Although neuroimaging studies have provided valuable insights, several factors contribute to inconsistent and conflicting results. Variations in research observations, differences in patient populations, and the varying sensitivity and specificity of different neuroimaging methods all play a role. These issues result in varied findings, thereby hindering the derivation of a definitive and reliable conclusion. In the future, research efforts in this domain should place a high priority on tackling these limitations. This will not only contribute to a more comprehensive understanding of the disease mechanism but also potentially lead to the development of more effective prevention and treatment strategies for HAND.

Research on HAND is impeded by methodological constraints, particularly the concentration on male subjects and the oversight of gender disparities in cognitive impairments, coupled with the underrepresentation of female participants. Studies suggest that female patients may suffer from more severe cognitive deficits and a higher incidence of neurocognitive disorders (198), necessitating more inclusive research that takes gender into account. Concurrently, the relationship between weight status and HAND is complex. An increase in weight suggests enhanced health and wellbeing for underweight PLWH, but worsening inflammation for overweight patients (199–201). This complexity highlights the necessity for future research to categorize PLWH based on weight, in order to clarify the effects of obesity on cognitive deterioration and brain health. In summary, to advance our comprehension of HAND, future research needs to tackle gender disparities, augment sample sizes, and delve into the sophisticated effects of weight on cognitive function and neural circuitry in HIV-infected populations.

Current research predominantly concentrates on the chronic phase of HIV, where patients have typically received cART and exhibit lower viral load. In a study by Cooley et al. (202), patients were categorized into three groups according to the viral load: virologic suppression (VS), low-level viremia, and virologic failure (VF). The study did not report significant differences in brain capacity or cognitive performance among the AIDS subgroups in a cross-sectional comparison. However, longitudinal data revealed that patients in the VF group showed reduced brain capacity and cognitive decline relative to the VS group, indicating that individuals in VF become more susceptible to impairments in brain structure and function. This highlights the necessity to study the effects of high viral load on the brains of PLWH and initiate the study upon acute HIV infection to catch the dynamic process of brain changes. Future efforts should be put into longitudinal research to figure out how fluctuations in viral load impact neurocognitive health at various stages of HIV.

Besides, the burgeoning field of imaging genomics, which synergies high-throughput imaging data—encompassing pathological histological images, MRI, and PET scans—with genomic information such as single nucleotide polymorphisms, DNA sequences, RNA expression, methylation, epigenetic markers, proteomics, and metabolomics data, holds the capability to bridge the gap between genetic composition and brain functionality (203, 204). This interdisciplinary methodology unveils the regulatory mechanisms by which specific genetic markers exert influence on the brain structure, function, and network organization. It also delves deep into the neural underpinnings and genetic bases of various brain disorders, providing a more comprehensive understanding of the complex interplay between genetics and neurological processes. While imaging genomics has been applied to conditions like Alzheimer's disease and schizophrenia, its potential in understanding HAND remains largely unexplored. In future research, the utilization of imaging genomics can be employed to conduct in-depth investigations into the influence of genes on the brain structure and function of individuals infected with HIV. This methodology will enable the identification of genes associated with cognitive decline and lay a solid foundation for the development of innovative targeted medications and therapeutic approaches. At present, the Allen Human Brain Atlas provides the most complete transcriptomic data of the brain cortex, leading to a spatial coupling analysis with brain MRI images and providing a new feasible way to investigate the driving factor of macroscale images (205). In summary, the application of imaging genomics in HAND holds promise for uncovering the genetic targets for diagnosis and therapeutic intervention.

In conclusion, neuroimaging advancements have significantly expanded our understanding of HAND, highlighting the need for further research to elucidate its complex pathogenesis and develop targeted interventions. Establishing high-quality databases and artificial intelligence platforms are possible choices to achieve breakthroughs at present. In the long run, a longitudinal cohort including but not limited to the neuroimaging data set will be necessary to further investigate the neural mechanisms of HAND, monitor disease progression, evaluate the efficacy of cART, and explore imaging markers for early diagnosis and therapeutic assessment of HAND.
